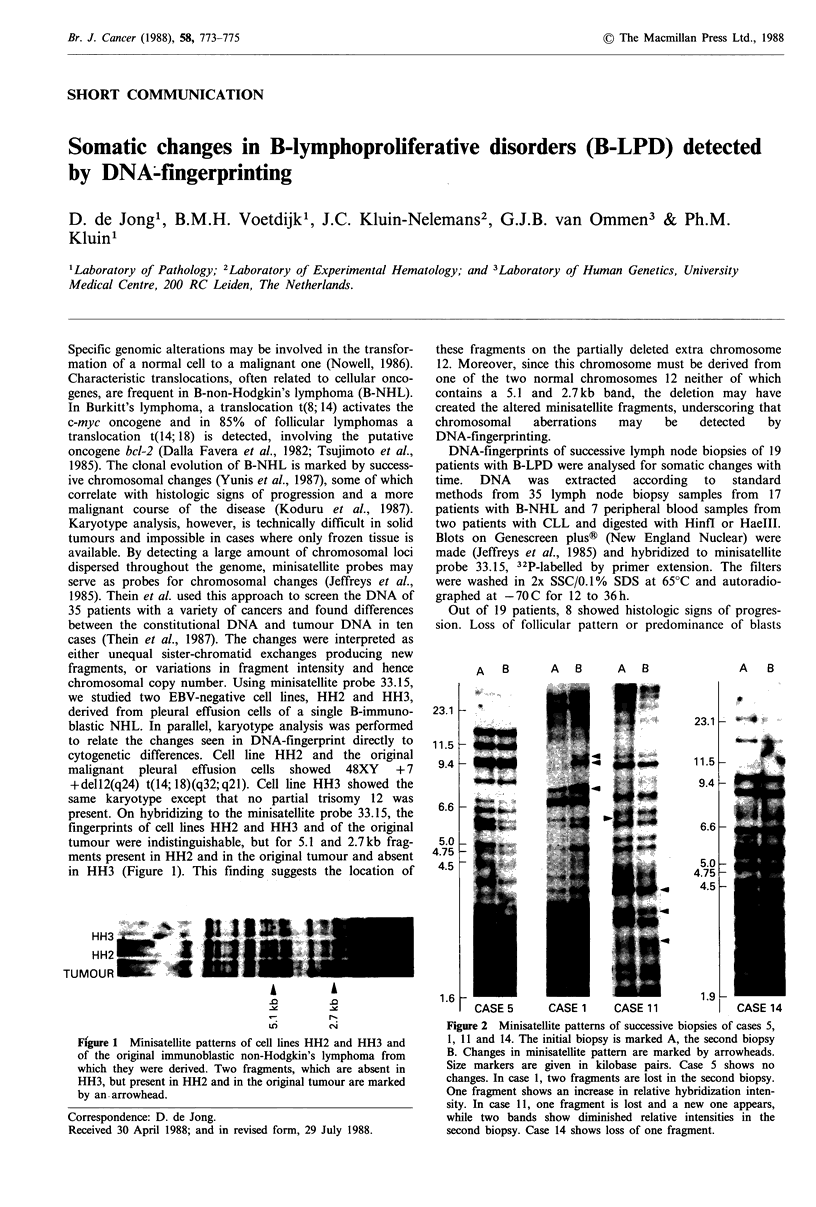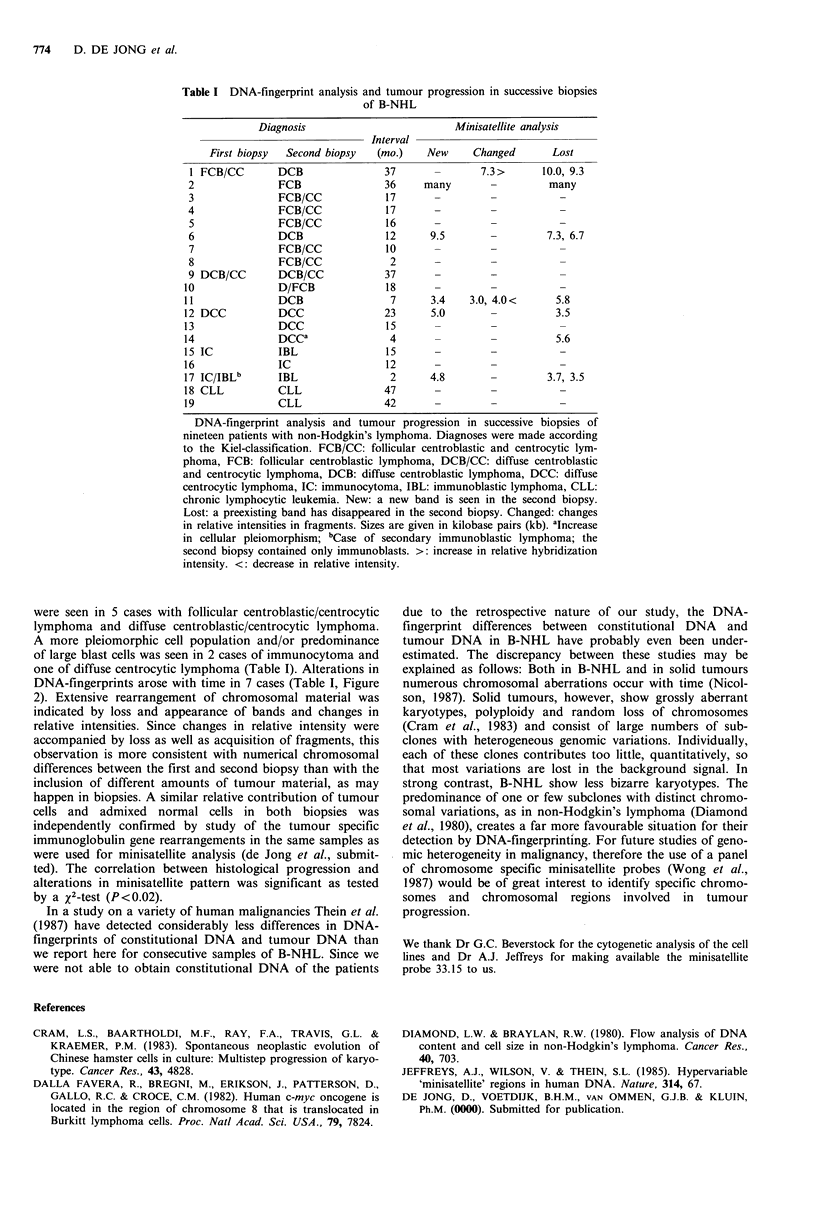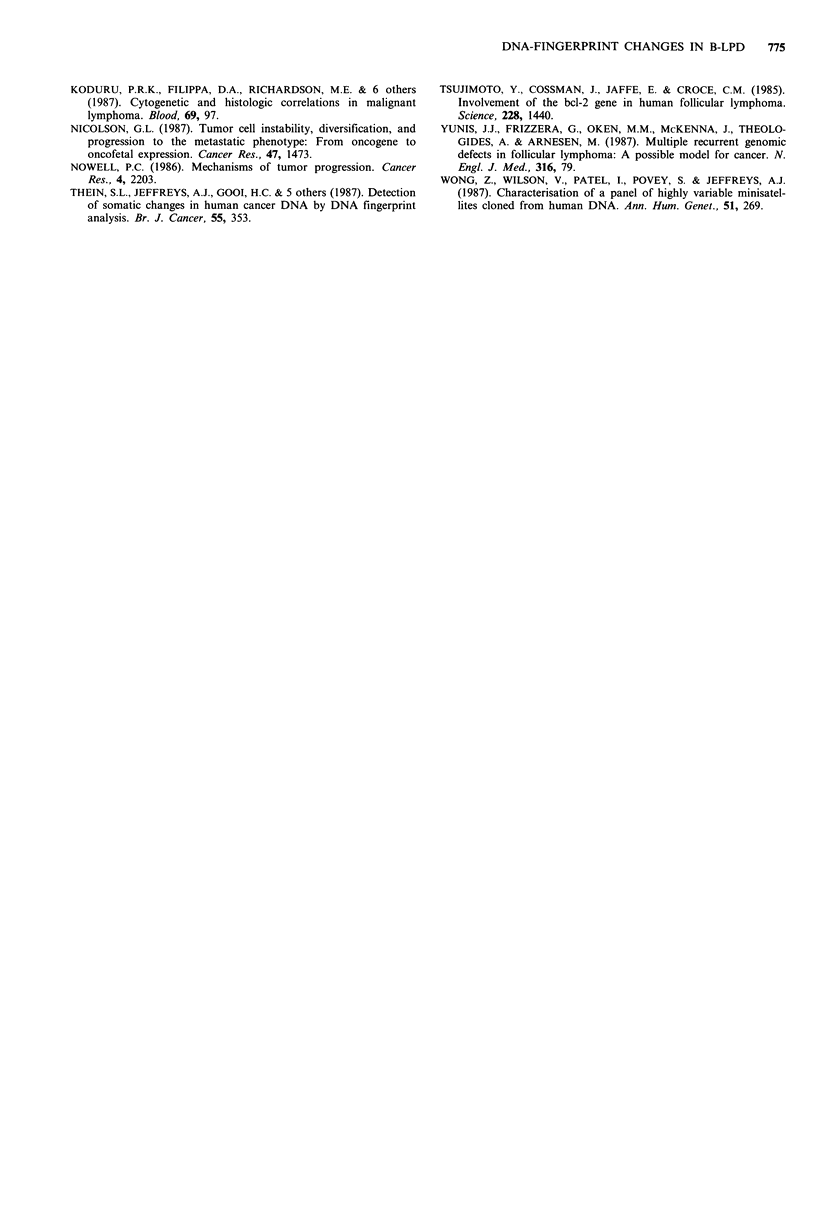# Somatic changes in B-lymphoproliferative disorders (B-LPD) detected by DNA-fingerprinting.

**DOI:** 10.1038/bjc.1988.306

**Published:** 1988-12

**Authors:** D. de Jong, B. M. Voetdijk, J. C. Kluin-Nelemans, G. J. van Ommen, P. M. Kluin

**Affiliations:** Laboratory of Pathology, University Medical Centre, Leiden, The Netherlands.

## Abstract

**Images:**


Br. J. Cancer (1988), 58, 773-775 © The Macmillan Press Ltd., 1988

SHORT COMMUNICATION

### Somatic changes in B-lymphoproliferative disorders (B-LPD) detected by DNA-fingerprinting

D. de Jong[1], B.M.H. Voetdijk[1], J.C. Kluin-Nelemans[2], G.J.B. van Ommen[3] & Ph.M. Kluin[1]

[1]*Laboratory of Pathology;* [2]*Laboratory of Experimental Hematology; and* [3]*Laboratory of Human Genetics, University Medical Centre, 200 RC Leiden, The Netherlands.*

Specific genomic alterations may be involved in the transformation of a normal cell to a malignant one (Nowell, 1986). Characteristic translocations, often related to cellular oncogenes, are frequent in B-non-Hodgkin's lymphoma (B-NHL). In Burkitt's lymphoma, a translocation t(8; 14) activates the c-*myc* oncogene and in 85% of follicular lymphomas a translocation t(14; 18) is detected, involving the putative oncogene *bcl-2* (Dalla Favera *et al.*, 1982; Tsujimoto *et al.*, 1985). The clonal evolution of B-NHL is marked by successive chromosomal changes (Yunis *et al.*, 1987), some of which correlate with histologic signs of progression and a more malignant course of the disease (Koduru *et al.*, 1987). Karyotype analysis, however, is technically difficult in solid tumours and impossible in cases where only frozen tissue is available. By detecting a large amount of chromosomal loci dispersed throughout the genome, minisatellite probes may serve as probes for chromosomal changes (Jeffreys *et al.*, 1985). Thein *et al.* used this approach to screen the DNA of 35 patients with a variety of cancers and found differences between the constitutional DNA and tumour DNA in ten cases (Thein *et al.*, 1987). The changes were interpreted as either unequal sister-chromatid exchanges producing new fragments, or variations in fragment intensity and hence chromosomal copy number. Using minisatellite probe 33.15, we studied two EBV-negative cell lines, HH2 and HH3, derived from pleural effusion cells of a single B-immunoblastic NHL. In parallel, karyotype analysis was performed to relate the changes seen in DNA-fingerprint directly to cytogenetic differences. Cell line HH2 and the original malignant pleural effusion cells showed 48XY +7 +del12(q24) t(14; 18)(q32; q21). Cell line HH3 showed the same karyotype except that no partial trisomy 12 was present. On hybridizing to the minisatellite probe 33.15, the fingerprints of cell lines HH2 and HH3 and of the original tumour were indistinguishable, but for 5.1 and 2.7 kb fragments present in HH2 and in the original tumour and absent in HH3 (Figure 1). This finding suggests the location of these fragments on the partially deleted extra chromosome 12. Moreover, since this chromosome must be derived from one of the two normal chromosomes 12 neither of which contains a 5.1 and 2.7 kb band, the deletion may have created the altered minisatellite fragments, underscoring that chromosomal aberrations may be detected by DNA-fingerprinting.

**Figure 1** Minisatellite patterns of cell lines HH2 and HH3 and of the original immunoblastic non-Hodgkin's lymphoma from which they were derived. Two fragments, which are absent in HH3, but present in HH2 and in the original tumour are marked by an arrowhead.

Correspondence: D. de Jong.
Received 30 April 1988; and in revised form, 29 July 1988.

DNA-fingerprints of successive lymph node biopsies of 19 patients with B-LPD were analysed for somatic changes with time. DNA was extracted according to standard methods from 35 lymph node biopsy samples from 17 patients with B-NHL and 7 peripheral blood samples from two patients with CLL and digested with HinfI or HaeIII. Blots on Genescreen plus® (New England Nuclear) were made (Jeffreys *et al.*, 1985) and hybridized to minisatellite probe 33.15, $^{32}P$-labelled by primer extension. The filters were washed in 2x SSC/0.1% SDS at 65°C and autoradiographed at −70C for 12 to 36h.

Out of 19 patients, 8 showed histologic signs of progression. Loss of follicular pattern or predominance of blasts

**Figure 2** Minisatellite patterns of successive biopsies of cases 5, 1, 11 and 14. The initial biopsy is marked A, the second biopsy B. Changes in minisatellite pattern are marked by arrowheads. Size markers are given in kilobase pairs. Case 5 shows no changes. In case 1, two fragments are lost in the second biopsy. One fragment shows an increase in relative hybridization intensity. In case 11, one fragment is lost and a new one appears, while two bands show diminished relative intensities in the second biopsy. Case 14 shows loss of one fragment.

**Table I** DNA-fingerprint analysis and tumour progression in successive biopsies of B-NHL

| | *Diagnosis* | | | *Minisatellite analysis* | | |
|---|---|---|---|---|---|---|
| | *First biopsy* | *Second biopsy* | *Interval (mo.)* | *New* | *Changed* | *Lost* |
| 1 | FCB/CC | DCB | 37 | – | 7.3> | 10.0, 9.3 |
| 2 | | FCB | 36 | many | – | many |
| 3 | | FCB/CC | 17 | – | – | – |
| 4 | | FCB/CC | 17 | – | – | – |
| 5 | | FCB/CC | 16 | – | – | – |
| 6 | | DCB | 12 | 9.5 | – | 7.3, 6.7 |
| 7 | | FCB/CC | 10 | – | – | – |
| 8 | | FCB/CC | 2 | – | – | – |
| 9 | DCB/CC | DCB/CC | 37 | – | – | – |
| 10 | | D/FCB | 18 | – | – | – |
| 11 | | DCB | 7 | 3.4 | 3.0, 4.0< | 5.8 |
| 12 | DCC | DCC | 23 | 5.0 | – | 3.5 |
| 13 | | DCC | 15 | – | – | – |
| 14 | | DCC[a] | 4 | – | – | 5.6 |
| 15 | IC | IBL | 15 | – | – | – |
| 16 | | IC | 12 | – | – | – |
| 17 | IC/IBL[b] | IBL | 2 | 4.8 | – | 3.7, 3.5 |
| 18 | CLL | CLL | 47 | – | – | – |
| 19 | | CLL | 42 | – | – | – |

DNA-fingerprint analysis and tumour progression in successive biopsies of nineteen patients with non-Hodgkin's lymphoma. Diagnoses were made according to the Kiel-classification. FCB/CC: follicular centroblastic and centrocytic lymphoma, FCB: follicular centroblastic lymphoma, DCB/CC: diffuse centroblastic and centrocytic lymphoma, DCB: diffuse centroblastic lymphoma, DCC: diffuse centrocytic lymphoma, IC: immunocytoma, IBL: immunoblastic lymphoma, CLL: chronic lymphocytic leukemia. New: a new band is seen in the second biopsy. Lost: a preexisting band has disappeared in the second biopsy. Changed: changes in relative intensities in fragments. Sizes are given in kilobase pairs (kb). [a]Increase in cellular pleiomorphism; [b]Case of secondary immunoblastic lymphoma; the second biopsy contained only immunoblasts. >: increase in relative hybridization intensity. <: decrease in relative intensity.

were seen in 5 cases with follicular centroblastic/centrocytic lymphoma and diffuse centroblastic/centrocytic lymphoma. A more pleiomorphic cell population and/or predominance of large blast cells was seen in 2 cases of immunocytoma and one of diffuse centrocytic lymphoma (Table I). Alterations in DNA-fingerprints arose with time in 7 cases (Table I, Figure 2). Extensive rearrangement of chromosomal material was indicated by loss and appearance of bands and changes in relative intensities. Since changes in relative intensity were accompanied by loss as well as acquisition of fragments, this observation is more consistent with numerical chromosomal differences between the first and second biopsy than with the inclusion of different amounts of tumour material, as may happen in biopsies. A similar relative contribution of tumour cells and admixed normal cells in both biopsies was independently confirmed by study of the tumour specific immunoglobulin gene rearrangements in the same samples as were used for minisatellite analysis (de Jong *et al.*, submitted). The correlation between histological progression and alterations in minisatellite pattern was significant as tested by a $\chi^2$-test ($P<0.02$).

In a study on a variety of human malignancies Thein *et al.* (1987) have detected considerably less differences in DNA-fingerprints of constitutional DNA and tumour DNA than we report here for consecutive samples of B-NHL. Since we were not able to obtain constitutional DNA of the patients due to the retrospective nature of our study, the DNA-fingerprint differences between constitutional DNA and tumour DNA in B-NHL have probably even been underestimated. The discrepancy between these studies may be explained as follows: Both in B-NHL and in solid tumours numerous chromosomal aberrations occur with time (Nicolson, 1987). Solid tumours, however, show grossly aberrant karyotypes, polyploidy and random loss of chromosomes (Cram *et al.*, 1983) and consist of large numbers of subclones with heterogeneous genomic variations. Individually, each of these clones contributes too little, quantitatively, so that most variations are lost in the background signal. In strong contrast, B-NHL show less bizarre karyotypes. The predominance of one or few subclones with distinct chromosomal variations, as in non-Hodgkin's lymphoma (Diamond *et al.*, 1980), creates a far more favourable situation for their detection by DNA-fingerprinting. For future studies of genomic heterogeneity in malignancy, therefore the use of a panel of chromosome specific minisatellite probes (Wong *et al.*, 1987) would be of great interest to identify specific chromosomes and chromosomal regions involved in tumour progression.

We thank Dr G.C. Beverstock for the cytogenetic analysis of the cell lines and Dr A.J. Jeffreys for making available the minisatellite probe 33.15 to us.

**References**

CRAM, L.S., BAARTHOLDI, M.F., RAY, F.A., TRAVIS, G.L. & KRAEMER, P.M. (1983). Spontaneous neoplastic evolution of Chinese hamster cells in culture: Multistep progression of karyotype. *Cancer Res.*, **43**, 4828.

DALLA FAVERA, R., BREGNI, M., ERIKSON, J., PATTERSON, D., GALLO, R.C. & CROCE, C.M. (1982). Human c-*myc* oncogene is located in the region of chromosome 8 that is translocated in Burkitt lymphoma cells. *Proc. Natl Acad. Sci. USA.*, **79**, 7824.

DIAMOND, L.W. & BRAYLAN, R.W. (1980). Flow analysis of DNA content and cell size in non-Hodgkin's lymphoma. *Cancer Res.*, **40**, 703.

JEFFREYS, A.J., WILSON, V. & THEIN, S.L. (1985). Hypervariable 'minisatellite' regions in human DNA. *Nature*, **314**, 67.

DE JONG, D., VOETDIJK, B.H.M., VAN OMMEN, G.J.B. & KLUIN, Ph.M. (**0000**). Submitted for publication.

KODURU, P.R.K., FILIPPA, D.A., RICHARDSON, M.E. & 6 others (1987). Cytogenetic and histologic correlations in malignant lymphoma. *Blood*, **69**, 97.

NICOLSON, G.L. (1987). Tumor cell instability, diversification, and progression to the metastatic phenotype: From oncogene to oncofetal expression. *Cancer Res.*, **47**, 1473.

NOWELL, P.C. (1986). Mechanisms of tumor progression. *Cancer Res.*, **4**, 2203.

THEIN, S.L., JEFFREYS, A.J., GOOI, H.C. & 5 others (1987). Detection of somatic changes in human cancer DNA by DNA fingerprint analysis. *Br. J. Cancer*, **55**, 353.

TSUJIMOTO, Y., COSSMAN, J., JAFFE, E. & CROCE, C.M. (1985). Involvement of the bcl-2 gene in human follicular lymphoma. *Science*, **228**, 1440.

YUNIS, J.J., FRIZZERA, G., OKEN, M.M., McKENNA, J., THEOLOGIDES, A. & ARNESEN, M. (1987). Multiple recurrent genomic defects in follicular lymphoma: A possible model for cancer. *N. Engl. J. Med.*, **316**, 79.

WONG, Z., WILSON, V., PATEL, I., POVEY, S. & JEFFREYS, A.J. (1987). Characterisation of a panel of highly variable minisatellites cloned from human DNA. *Ann. Hum. Genet.*, **51**, 269.